# Design of a Horizontal Penetrometer for Measuring On-the-Go Soil Resistance

**DOI:** 10.3390/s101009337

**Published:** 2010-10-18

**Authors:** Mehmet Topakci, Ilker Unal, Murad Canakci, Huseyin Kursat Celik, Davut Karayel

**Affiliations:** 1 Department of Agricultural Machinery, Faculty of Agriculture, Akdeniz University, Antalya 01330, Turkey; E-Mails: mcanakci@akdeniz.edu.tr (M.C.); hkcelik@akdeniz.edu.tr (H.K.C.); dkarayel@akdeniz.edu.tr (D.K.); 2 Bucak Hikmet Tolunay Vocational School, Mehmet Akif Ersoy University, Burdur 15100, Turkey; E-Mail: ilkerunal@mehmetakif.edu.tr (I.U.)

**Keywords:** horizontal penetrometer, GPS, precision agriculture, mapping

## Abstract

Soil compaction is one of the main negative factors that limits plant growth and crop yield. Therefore, it is important to determine the soil resistance level and map it for the field to find solutions for the negative effects of the compaction. Nowadays, high powered communication technology and computers help us on this issue within the approach of precision agriculture applications. This study is focused on the design of a penetrometer, which can make instantaneous soil resistance measurements in the soil horizontally and data acquisition software based on the GPS (Global Positioning System). The penetrometer was designed using commercial 3D parametric solid modelling design software. The data acquisition software was developed in Microsoft Visual Basic.NET programming language. After the design of the system, manufacturing and assembly of the system was completed and then a field experiment was carried out. According to the data from GPS and penetration resistance values which are collected in Microsoft SQL Server database, a Kriging method by ArcGIS was used and soil resistance was mapped in the field for a soil depth of 40 cm. During operation, no faults, either in mechanical and software parts, were seen. As a result, soil resistance values of 0.2 MPa and 3 MPa were obtained as minimum and maximum values, respectively. In conclusion, the experimental results showed that the designed system works quite well in the field and the horizontal penetrometer is a practical tool for providing on-line soil resistance measurements. This study contributes to further research for the development of on-line soil resistance measurements and mapping within the precision agriculture applications.

## 1. Introduction

The tractors, tillage tools and the machine systems which are used in the agricultural production can cause field traffic. Especially today’s machines such as powerful tractors, combine harvesters *etc*. which are becoming heavier because of their additional attached equipment, have become a reason for high level of soil compaction observed in agricultural fields. Another reason for soil compaction is tillage in non-suitable terms of the soil. In addition to these external effects, natural effects such as excessive rainfall and drought can also be a reason for high levels of soil compaction [1–3].

Soil compaction can be defined as a function of the specific weight and humidity of the soil. During compaction, soil particles get closer each other and a diminishing of the entrapped air is seen. As a result; an increase is seen for soil bulk density and soil penetration resistance [4,5]. Soil compaction has also a negative effect on the physical, chemical and biological properties of the soil. This negative effect limits roots growth and the plants cannot complete their growth properly. Hence, less yield and economic losses are seen. In addition to this, the machines, which are operating on the compacted soil need extra energy [6,7]. Therefore, the determination of the soil penetration resistance level is quite important for sustainable production, yield and conservation of the farmland. It also has a place in the precision farming approach, which promises that the field performance could be tracked, mapped and analyzed down to the square meter level so that farmers can know how well or poorly each part of a field is producing [8].

In the scientific literature, two different methods are used for measuring soil penetration resistance. In the first method, soil samples are taken over the field at a certain depth of soil with the help of an open-ended pipe and then the samples are analysed in the laboratory to determine the penetration resistance. On the other method, a specifically sized conical tip is immersed into the ground vertically. The cone is sunk into the ground at a standard rate, starting at the soil surface of the soil [9]. The tip angle of the cone is 30°. Soil penetration resistance is calculated by dividing the force needed to insert the cone into the ground at a standard speed of 30 mm/s by the base area of the cone. This calculated value of the cone is called the cone index (CI) and is expressed by Equation (1) [10]:

(1) CI=F/A.

where CI: Cone index [MPa], F: Force [N], A= Base area [mm^2^].

To determine the cone index of the soil, cone penetrometers are used. Generally, static and dynamic penetrometers are used in the applications. The cone acts perpendicular to the surface in both methods. The difference between the two methods lies in how the force to insert the cone into soil is provided.

In the static method, the cone is pushed into the ground. To achieve this mechanical, hydraulic or electrical power can be used. To measure and record the index a load cell system located between support bar and the cone is used [11].

Nowadays communication technologies and developments in the precision agriculture applications field allow researchers to measure soil properties instantaneously and dynamically [12]. Much of the research in the scientific literature indicates the importance of the determination of the soil resistance levels instantaneously and mapping of the soil properties. To achieve this, there is a focus on the design of horizontal penetrometers, which can make instantaneous measurements, using GPS systems, and field mapping operations. The publications also indicate that the usage of instantaneous measurement techniques, equipment, GPS systems and soil maps of the soil properties have an important role on raising of the yield and allowing faster and easier field processes [7,13–18].

In this study, a system was designed to determine the soil resistance level instantaneously and assist with field mapping. As a new product, a horizontal penetrometer with a cone, which has an integrated load cell, was designed using the SolidWorks 3D parametric solid modelling design software. In the system, a data acquisition system based on GPS was utilised and special software which can transfer the files in desired formats to GIS mapping programs was developed for field mapping operations. In the field experiments, the system was operated with a tractor. All data from the penetrometer and the GPS were collected in a notebook in the operation and the related results and maps are presented.

## 2. Materials and Methods

### 2.1. Design of the System

**Figure 1.**
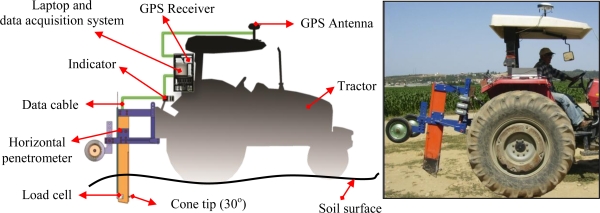
Structure of the designed system.

The main aim of the designed system is to measure the horizontal soil resistance and map it. The system involves three main components (Figure 1):

- Mechanical system: It is attached to tractor with three point mounted implements and it moves horizontally in the soil. The load cell is attached to the system to measure soil penetration resistance instantaneously.
- Data acquisition system: The system is used to collect and process the data from a GPS receiver and the load cell on the mechanical system for mapping operations.
- Software: The software is prepared to process the data and convert it to a desired file format for GIS (Geographical Information Systems) programs.

#### 2.1.1. Mechanical System

**Figure 2.**
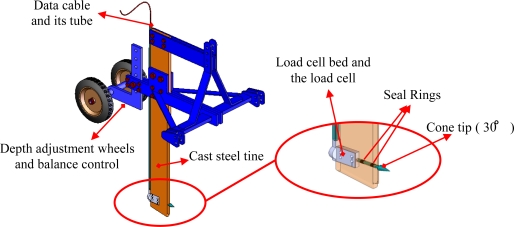
Design of the mechanical system.

The mechanical system is shown in Figure 2. The tine of the horizontal penetrometer was made of steel. The load cell bed was formed into the steel tine. The load cell was placed in this section and the top was covered. This prevents possible damage to the load cells under the soil. The data cables of the load cells were transported to the data collection unit by being passed through the metal tubes placed behind the penetrometer’s tine. Afterwards, into the hole, a conical-shaped tip of 30° was placed and fixed to the load cell. The surface of the designed conical-shaped tip is 706.5 mm^2^. Insulation seals were used in order to prevent probable leakage of water or soil particles into the holes on the body. An adjustable wheel system was placed behind the penetrometer to ensure a well-maintained depth adjustment and proper running of the machine. The total weight of the designed mechanical system was 310 kg.

#### 2.1.2. Data Acquisition System

**Figure 3.**
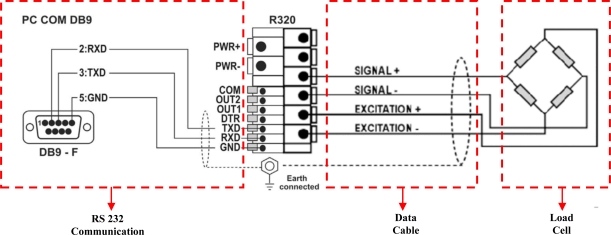
Load cell, indicator and RS232 communication system.

To measure the soil resistance, an S type 500 kg capacity H3-C3 (Zemic Europe B.V., Leerlooierstraat, NL) load cell was used. The load cell has a value of 2 ± 0.004 mV/V rated output and 0.01% accuracy of measurement. An R320 indicator (Rinstrum Pty Ltd, Brisbane, Australia) was used for the purpose of increasing the signal produced by the load cell and converting it into a strength value after the digital signal conversion process. The indicator has a circuit ratio of 20 Hz and a 24 bit sigma-delta ADC (Analog Digital Converter). Calibration of the load cell was performed via at connected indicator, according to the datasheet instructions provided. The range networking and indicator connection of the load cell is shown in Figure 3. The strength value which was measured through the RS232 serial networking port was sent to the serial port of the laptop within the system. Connection speed was set to 9,600 baud.

**Figure 4.**
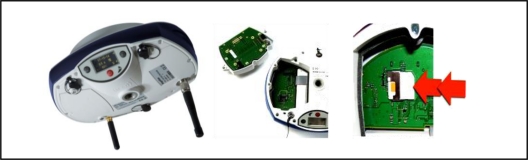
Promark500 GPS receiver and installing SIM card.

A Promark 500 GPS (Magellan Co., Santa Clara, CA, USA) receiver was used in the system to map the soil resistance. The receiver has 75 channels and up to 20 Hz data output rate. It is the most flexible GNSS (Global Navigation Satellite Systems) surveying system available, offering multiple operating modes, configurations and communication modules (UHF, GSM/GPRS, EDGE) and protocols. It can be connected to Corse-TR (Continuously Operating Reference Stations-Turkey) via a phone data card (SIM Card) to receive correction signals (Figure 4).

The receiver has a 99% confidence interval. The accuracy of the position obtained varies (according to the manufacturer’s data) from below 10 mm with correction signals. The receiver has RS-232, Bluetooth and USB ports to allow National Marine Electronics Association (NMEA) 0183 data transfer to a laptop computer. The data such as the geographical coordinates and advance speed of the spot that is being measured were then sent to the serial port of the laptop. The RS-232 serial communication protocol was used for two way communications between the GPS receiver and the laptop using serial data cable of the GPS receiver. Ther GPS receiver installation data was sent to set up the GPS receiver via the software. At the same time, NMEA (National Marine Electronics Association) 0183 row data were transferred to laptop by the GPS receiver. The communication speed was set at 9,600 baud.

#### 2.1.3. The Software

**Figure 5.**
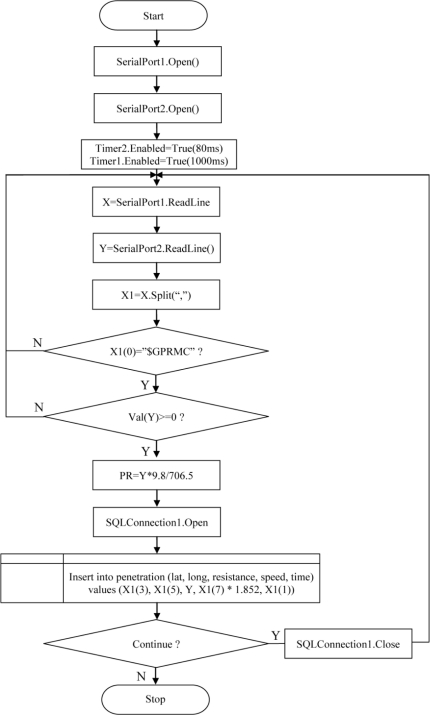
Flow chart of the software.

Software was written in Visual Basic.NET programming language (Microsoft Corp., Redmond, WA, USA) to process the load cell indicator and GPS receiver data. Serial port and timer objects in the Visual Basic.NET toolbox were used to read the RS-232 communication port. The serial port object is used to send and receive data to and from the load cell indicator and GPS receiver. The timer object is used to read the serial port of the laptop for specific time intervals. Two timers were used within the software. The first timer was set to read the force data sent by the indicator at intervals of 80 ms. Then the second timer was set to read the data sent by the GPS receiver, at intervals of 1,000 ms. The flow chart of the designed software is shown in Figure 5 above.

In the system, GPS receivers send data such as latitude, longitude, speed, time, *etc*. by cable to other electronic devices via the RS-232 serial port. NMEA 0183 is a standard protocol, used by GPS receivers to transmit data. NMEA 183 sentences are all American Standard Code for Information Interchange (ASCII). Each sentence begins with dollar sign ($) and the next five characters determine the sentence type. The “$GPRMC” sentence type is the most important and useful format and includes positional and temporal data. For this reason, this sentence type was used in the study. The GPS receiver sends data every second to the software. “$GPRMC” sentence fields are separated with commas. In the first step the sentence is stored in an array variable by the software; in the second step sentence fields are split by a Split command in the software and in the third step sentence fields are stored to the SQL Server 2005 database.

Latitude and longitude data are received from a GPS receiver in the NMEA-0183 format in ddmm. Mmmm units, where dd equals degrees, *mm* equals minutes, and *mmmm* is decimal minutes. For many purposes, positional information in this format is more than adequate. However, when plotting position information on maps or carrying out supplemental calculations using the position coordinates, it can be advantageous to work instead with the corresponding grid coordinates on a particular map projection.

**Figure 6.**
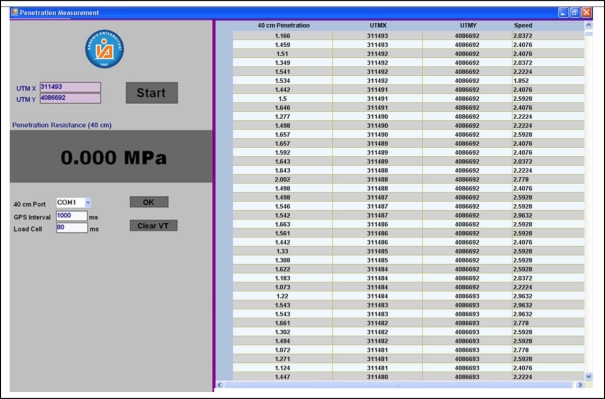
Developed software for penetration measurement.

One of the most widely used map projection and grid systems is the Universal Transverse Mercator (UTM) system. UTM grid coordinates are related to geodetic coordinates, and indicates the corrections to be applied to grid distance and bearings to get the actual true quantities on the Earth’s surface. For this reason, data that received from GPS receiver was converted to UTM format, and stored to the database by the software. The interface of the developed software is shown in Figure 6.

The signals from load cell were converted to force data by the indicator. Then the force data was sent through the serial port by the indicator using two different series data formats, given in Table 1 [19].

Format 1 was employed in order to send the force value to the software. The software interprets the format that is on the serial port of the laptop as an 11 character-long data string. Within this data string, the values 3 to 9 are processed and labelled as force data by the software itself. For the obtained force data to be converted into penetration resistance (PR), the use of Equation 2 was preferred:

(2) $$\mathrm{PR}(\mathrm{MPa})=\frac{\mathrm{Force}(N)}{\mathrm{Cone Area}({\mathrm{mm}}^{2})}$$

### 2.2. Experimental Site

The field experiments using the system were carried out in agricultural research area of Akdeniz University. The research area is located approximately 20 km from Antalya between the coordinates of 30.84 E and 36.94 N. The soil type is clay-loam and consists of 41% sand, 26% silt, 33% clay. Content of organic matter was 1.3%. Soil bulk density, water content and soil resistance values were determined as 1.32 g/cm^3^, 7.5%, and 1.45 MPa at a depth between 0 and 20 cm, and 1.38 g/cm^3^, 8.9%, 1.89 MPa at a depth between 20 and 40 cm, respectively.

### 2.3. Data Collection

The experimental field is 20 ha in size. The designed system was connected to a Massey Ferguson 3095D four-wheeled tractor. During the experiments, some small variations were seen in the tractor speed, even though care was taken to keep the tractor speed at a constant value to avoid any negative effect of speed changes on the penetration resistance. The experiments were carried out in a field shortly after a wheat harvest and measurement values of 40 cm operation depth and 15 m linear intervals were obtained. Much research indicates that the depth of the hard pan is mostly between 30 and 60 cm. The depth of 40 cm has been chosen as working depth to get data on the hard pan level of the field. The average speed of 2.39 km h^−1^ was calculated according to data from the GPS receiver. Forward speeds of 1.80 km h^−1^ and 2.96 km h^−1^ were determined as the minimum and maximum values, respectively. The time interval for the entire measurement was set to 1 second and 5,025 data points were stored in the database.

## 3. Results and Discussion

**Figure 7.**
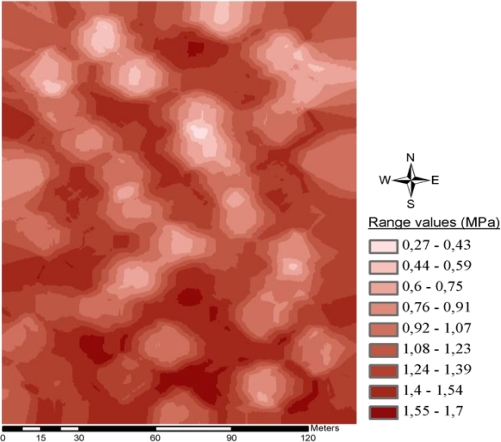
Soil Resistance map for 40 cm depth in soil.

**Figure 8.**
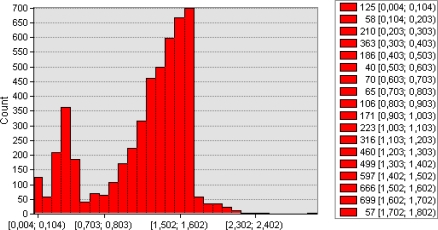
Histogram of 40 cm soil resistance values (MPa).

During the experiment within the 20 ha field, geographical coordination and progress values for 5,025 spots and soil resistance values for 40 cm depth soil penetration resistance values were collected. Measured minimum and maximum soil resistance values were 0.2 and 3 MPa, respectively. No faults were detected either in the mechanical or software parts of the system during operation. The obtained data was then stored in a format adaptable to the mapping programs in the Microsoft SQL Server 2005 database. The database was transformed into the ArcGIS 9.3 mapping software. For the creation of the map, ordinary kriging interpolation was applied. Figure 7 illustrates soil penetration resistance of 40 cm depth in the experimental field. A histogram of the soil penetration resistance values is given in Figure 8.

Similar penetrometers were developed and tested by Alihamsyah *et al*. [20], Bolenius *et al*. [18] and Andrade *et al*. [21,22]. According to Adamchuk *et al*. [12], data collected with online soil resistance measurement systems is potentially useful. For example, adjusting tillage depth to remove the hard pan has a potential economic impact and may be become commercially available soon.

In addition to these results, for the obtained penetration resistance values to be more meaningful, moisture content and bulk density of the soil should be measured instantly as well. This instantaneous measurement of the moisture content and bulk density of the soil are planned in the next step of this study. To achieve this the system was also designed to be upgradable.

## 4. Conclusions

In this paper a new design for instantly measuring the soil resistance and its mapping has been discussed. The starting point of this research is that unlike a vertical penetrometer, a horizontal one offers more data on the field, so the high volume of data at hand provides more opportunity to make active, effective and productive evaluations about the field type. From the study, it can be concluded that one of the most crucial factors during the data collection is ‘speed progress’. During the experimental study, speed progress must to be kept as constant as possible.

Maps are regarded as tools for processing coordinate data and also for data analysis and representation. Another important factor of the maps is their contribution in aiding users in making quick and reasonable decisions, for which the quality of the data gains importance. In this respect, the high number of penetration resistance values obtained from the study improves the map quality.

The new database which was created with the help of the software can be regarded as a core for different mapping software. Additionally, this soil penetration resistance map can be a source for delicate agricultural applications across different fields. A deficiency has been detected in terms of the lack of instant moisture level measurements within the system. Further studies will adapt this instant soil moisture content system and so the interpretation of the data will be verified and improved.

## Figures and Tables

**Table 1.** Serial Data Format for R320 indicator.

**Format**	**Description**
Format 1	<STX><SIGN><WEIGHT(7)><STATUS><ETX>
Format 2	<STX>SIGN<WEIGHT7><S1><S2><S3><S4><UNITS(S)><ETX>

where:
STX: Start of transmission character (ASCII 02). ETX: End of transmission character (ASCII 03). SIGN: The sign of the weight reading (space for positive, dash (-) for negative). WEIGHT(7): A seven character string containing the current weight including the decimal point. If there is no decimal point, then the first character is a space. Leading zero blanking applies. STATUS: Provides information on the weight reading. The characters G/N/U/O/M/E represent Gross/Net/Underload/Overload/Motion/Error, respectively. UNITS(3): A three character string, the first character being a space, followed by the actual units (eg. ^kg or ^^t). If the weight reading is not stable, the unit string is sent as ^^^. S1: Displays G/N/U/O/E representing Gross/Net/Underload/Overload/Error, respectively. S2: Displays M/^ representing Motion/Stable, respectively. S3: Displays Z/^ representing centre of Zero/Non-Zero, respectively. S4: Displays—representing single range.
